# Temporospatial shifts within commercial laboratory mouse gut microbiota impact experimental reproducibility

**DOI:** 10.1186/s12915-020-00810-7

**Published:** 2020-07-03

**Authors:** Rabindra K. Mandal, Joshua E. Denny, Morgan L. Waide, Qingsheng Li, Neal Bhutiani, Charles D. Anderson, Becca V. Baby, Venkatakrishna R. Jala, Nejat K. Egilmez, Nathan W. Schmidt

**Affiliations:** 1 Department of Microbiology and Immunology, University of Louisville, Louisville, KY 40202 USA; 2 Present Address: Ryan White Center for Pediatric Infectious Diseases and Global Health, Department of Pediatrics, Indiana University School of Medicine, 1044 W. Walnut St., Indianapolis, IN 46202 USA

**Keywords:** Temporospatial shift, Gut microbiota, Malaria, Lung tumorigenesis, Salmonellosis, Colitis

## Abstract

**Background:**

Experimental reproducibility in mouse models is impacted by both genetics and environment. The generation of reproducible data is critical for the biomedical enterprise and has become a major concern for the scientific community and funding agencies alike. Among the factors that impact reproducibility in experimental mouse models is the variable composition of the microbiota in mice supplied by different commercial vendors. Less attention has been paid to how the microbiota of mice supplied by a particular vendor might change over time.

**Results:**

In the course of conducting a series of experiments in a mouse model of malaria, we observed a profound and lasting change in the severity of malaria in mice infected with *Plasmodium yoelii*; while for several years mice obtained from a specific production suite of a specific commercial vendor were able to clear the parasites effectively in a relatively short time, mice subsequently shipped from the same unit suffered much more severe disease. Gut microbiota analysis of frozen cecal samples identified a distinct and lasting shift in bacteria populations that coincided with the altered response of the later shipments of mice to infection with malaria parasites. Germ-free mice colonized with cecal microbiota from mice within the same production suite before and after this change followed by *Plasmodium* infection provided a direct demonstration that the change in gut microbiota profoundly impacted the severity of malaria. Moreover, spatial changes in gut microbiota composition were also shown to alter the acute bacterial burden following *Salmonella* infection, and tumor burden in a lung tumorigenesis model.

**Conclusion:**

These changes in gut bacteria may have impacted the experimental reproducibility of diverse research groups and highlight the need for both laboratory animal providers and researchers to collaborate in determining the methods and criteria needed to stabilize the gut microbiota of animal breeding colonies and research cohorts, and to develop a microbiota solution to increase experimental rigor and reproducibility.

## Background

Although the self-correcting nature of science is a virtue, recent years have experienced a growing awareness and concern across diverse scientific disciplines that published studies are not always reproducible [1, 2]. Lack of reproducibility slows the advancement of science, erodes public confidence in science, and impedes the development of new therapeutics [1–3]. A *Nature* survey of 1576 scientists identified that 70% of researchers have failed to reproduce a published result [2]. A number of reports and editorials from journals, including *Science* and *Nature* and the National Institutes of Health, have identified procedures to improve reproducibility [3–6]. The *Nature* survey found that respondents were less inclined to attribute variability in reagents as a contributor to irreproducibility [2]. Yet, there is a growing recognition that mouse studies that fail to control for differences in murine gut microbiota are indeed factors that impact reproducibility of mouse studies [7–9].

Differences in gut microbiota among genetically similar mice obtained from different commercial vendors were originally reported 30 years ago [10]. This report provided a basic characterization of differences in gut bacteria without any association to a biological effect. As our appreciation of the effect of gut microbiota on host physiology has rapidly grown over the previous decade, so have the number of publications that identified differences in gut microbiota among genetically similar mice from different vendors and that these differences can have profound impacts on various biological processes [11–20]. Moreover, genetically identical mice within the same institution can have differences in gut microbiota that alter host biology [21–24].

In contrast to the known differences in gut bacteria in mice from different vendors and genetically identical mice within the same institution, there is much less known regarding variation in gut bacteria from a specific vendor over time. In one study that sampled fecal pellets from a total of forty-six 129X1/SvJ mice over time and among four separate shipments from Jackson Laboratories, only 18% of the observed variation in bacteria populations among all samples was explained by shipment groups [25]. Indeed, taxonomic plots at the phylum level showed similar taxonomic abundances between shipments, with the greatest variability observed between individual mice independent of the shipment group. Although variation between shipments was noted, these were not temporally consistent differences (i.e., shipments I and III were similar to one another while shipments II and IV were similar to one another). To our knowledge, the study by Hoy and colleagues [25] is the only published data regarding variation in gut bacteria in mice shipped from the same vendor over time.

In our efforts to understand how gut microbiota impacts the severity of malaria, we utilized our observation that mice purchased from different vendors display relatively low or high *Plasmodium* parasite burdens [18, 26–28]. Mice purchased from Taconic Biosciences were consistently used as a source of mice with low parasite burden. Curiously, we noted a stark change in parasite burden in Taconic mice, despite ordering from the same production room, which created considerable disruption to ongoing experiments. In this study, we demonstrate that a marked change in gut bacteria occurred within a defined vendor production suite that impacted experimental reproducibility in two infectious disease models and a murine tumorigenesis model. These observations raise the possibility that other investigators may have observed similar deviations within their mouse model systems. Furthermore, this is a demonstration that mouse gut microbiota, whether from commercial vendors or in-house sources, are an important factor to consider for reproducible, controlled results. Finally, these results emphasize the need for researchers to fully understand that variability is possible even when animals are purchased from the same room at the same vendor, and the need for commercial vendors to educate their customers about their husbandry practices and health standards. Ultimately, researchers and vendors should come together to determine the level of control and reporting that will aid in efforts to account for potential microbiota effects.

## Results

### Change in the severity of malaria within Taconic mice correlates with shift in gut bacteria populations

**Fig. 1:**
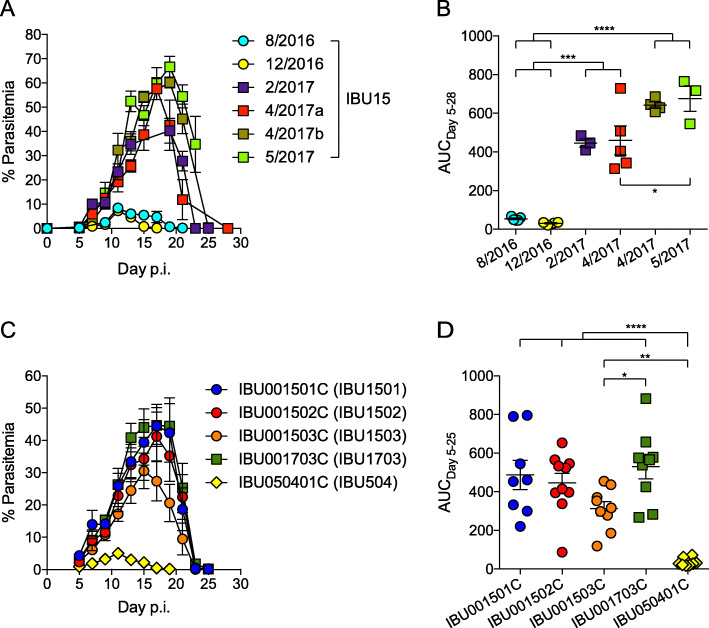
Temporally discrete *P. yoelii* parasitemia profiles in Taconic C57BL/6N mice. C57BL/6N mice from Taconic Biosciences were infected with *P. yoelii* 17XNL followed by the analysis of parasite burden. **a** Percent parasitemia was measured in Taconic mice obtained from a single C57BL/6N production suite, Isolated Barrier Unit™ (IBU15), on the indicated date. Data are from 3 to 5 mice per group.. **b** Area under the parasitemia curve (AUC) from **a** with each symbol representing an individual mouse. **c** Percent parasitemia in Taconic mice obtained from specific C57BL/6N IBUs. Data are cumulative results with 8–10 mice per group from two experiments. **d** AUC from **c** with each symbol representing an individual mouse. Data are mean ± S.E. **b**,**d**. One-way ANOVA with Tukey’s multiple comparisons test. *p < 0.05, **p < 0.01, ***p < 0.001, ****p < 0.0001

In an effort to avoid variation in gut microbiota, our laboratory has specifically ordered C57BL/6NTac mice from Taconic Biosciences from the production room Isolated Barrier Unit™ (IBU)15 located at the Germantown, New York facility. Between the years of 2012 and 2016, these mice displayed a relatively low parasite burden following infection with *Plasmodium yoelii* 17XNL, with peak parasitemia reaching 10–20% and clearance of the parasite within 3 weeks post-infection (Fig. 1a, b, and as published [18, 26]). Surprisingly, between December 2016 and February 2017, there was a profound change in the parasitemia profile of these mice, where peak parasitemia in mice obtained from IBU15 post-2017 consistently reached 50–60% and clearance of parasite occurred around 4 weeks post-infection (Fig. 1a, b). In an effort to understand what led to this change, it was discovered that the IBU15 production room is a suite of three rooms (IBU001501C, IBU001502C, and IBU001503C). As it was unknown that IBU15 was a suite of rooms at the time the experiments were conducted, mice in Fig. 1a cannot be distinguished between IBU001501C, IBU001502C, and IBU001503C. There is also a separate C57BL/6N production room (IBU001703C) at the Germantown, New York facility. Taconic Biosciences also has a C57BL/6N production room (IBU050401C) in Cambridge City, Indiana. C57BL/6N mice from each of these five separate rooms were obtained to determine if mice displayed similar *P. yoelii* parasite burdens. Following *P. yoelii* infection, mice from each of the four rooms from the Germantown, New York facility displayed relatively high parasite burdens peaking between 30 and 50% with clearance of *P. yoelii* occurring between 3 and 4 weeks post-infection (Fig. 1c, d). In contrast, mice from the Cambridge City, Indiana facility (IBU050401C) exhibited peak parasitemia of approximately 5% and cleared the parasite about 2 weeks post-infection (Fig. 1c, d). Hereafter, IBU001501C, IBU001502C, IBU001503C, IBU001703C, and IBU050401C will be shortened as IBU1501, IBU1502, IBU1503, IBU1703, and IBU504, respectively. Although these data did not explain the temporal change in malaria severity in IBU15 mice, they did demonstrate a consistent phenotype in mice from multiple rooms from the Germantown, New York facility.

**Fig. 2:**
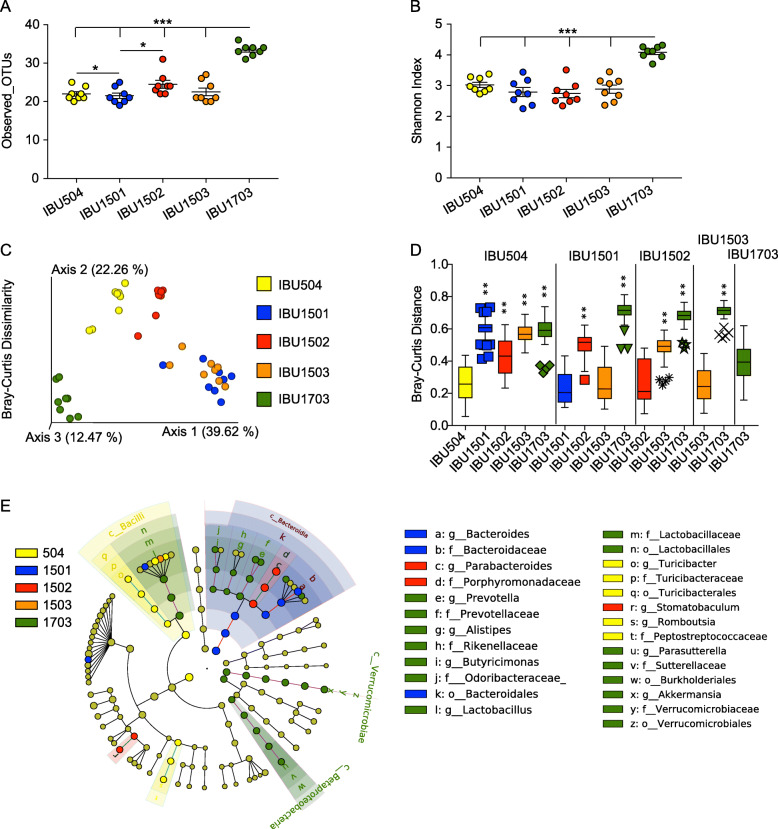
Discrete parasitemia profiles correlate with a shift in Taconic C57BL/6N gut bacteria populations within a defined production suite. Fecal pellets were collected from mice (*n* = 8 mice; 4 mice each from two separate shipments) obtained from the indicated Taconic IBUs and subjected to 16S rRNA gene sequencing. Data were analyzed at species level with a 23,000 sequencing depth per sample. **a**, **b** Alpha diversity analysis using Observed_OTUs (**a**) and Shannon index (**b**). Data (mean ± S.E.) were analyzed by the Kruskal-Wallis test. **c**, **d** The PCoA plot shows beta diversity using Bray-Curtis dissimilarity distance (**c**), and the box and whisker plot shows their statistical significance by pairwise PERMANOVA with 999 permutations between IBUs on the top of vertical columns to IBUs on the X-axis (**d**). The box end depicts the lower and upper quartiles and the horizontal line inside the box is the median while points outside the whisker are outliers. The *Y*-axis shows the Bray-Curtis dissimilarity distance of IBUs on the *X*-axis to IBUs on the top of vertical columns. e Cladogram shows differentially abundant bacterial taxa among different IBUs with respective node color identified using LEfSe analysis. The cutoff for the LEfSe method was *p* < 0.05 (Kruskal-Wallis test) with LDA score > 4. *p < 0.05, **p < 0.01, ***p < 0.001

We previously demonstrated that distinct gut microbiota communities from different mouse vendors impact the severity of malaria [18]. This led to the hypothesis that there was a change in gut microbiota in mice from the IBU15 suite between December 2016 and February 2017. To first test this hypothesis, 16S rRNA gene sequencing was performed on fecal pellets obtained from mice ordered from each of the five separate IBUs to determine if differences in gut bacterial communities correlated with differences in *P. yoelii* infection. Alpha diversity measured with observed taxonomic units (OTUs) at the species level (total number of OTUs within a given sample) and Shannon index (considers both the number of OTUs and the evenness of those OTUs within the sample) showed that IBU1703 was significantly different from the other IBUs (*p* < 0.001; Fig. 2a, b). Similarly, observed OTUs were significantly different between IBU504 vs. IBU1501 and IBU1501 vs. IBU1502 (*p* < 0.05) (Fig. 2a). Additionally, beta diversity (i.e., a comparison of bacteria populations between samples) measured with Bray-Curtis dissimilarity index (measures both species richness and their abundance) and weighted UniFrac distance (measures species richness, their phylogenetic relatedness and abundances) showed that all the IBUs were significantly different from each other except IBU1501 and IBU1503 (*p* < 0.01; Fig. 2c, d and Additional file 1, Fig. S1E-F, respectively). However, Jaccard distance (measures species richness but not their abundance) and unweighted UniFrac distance (measures species richness and their phylogenetic relatedness but does not consider abundances of the species) showed that all the IBUs, including IBU1501 and IBU1503, were significantly different from each other (*p* < 0.01 for all comparisons except unweighted UniFrac distance IBU1502 vs. IBU1503, *p* < 0.05; Additional file 1, Fig. S1A-D). Thus, with the exception of when species abundance is taken into consideration between IBU1501 and IBU1503 (Fig. 1d), the data demonstrate that bacteria communities are different among these barrier rooms. Interestingly, multiple bacterial taxa were differentially abundant among the IBUs at different taxonomic levels (Fig. 2e). Most of the differentially abundant bacteria were present in IBU1703 (Fig. 2e). These data support the hypothesis, but only show a correlation between bacteria communities and *P. yoelii* parasite burden. Moreover, as these data are from mice obtained post-2016, they do not provide a direct demonstration that there was indeed a change in gut bacteria populations in IBU15 mice between December 2016 and February 2017.

### Shift in gut microbiota within a Taconic IBU caused profound alterations in the severity of malaria in mice

**Fig. 3:**
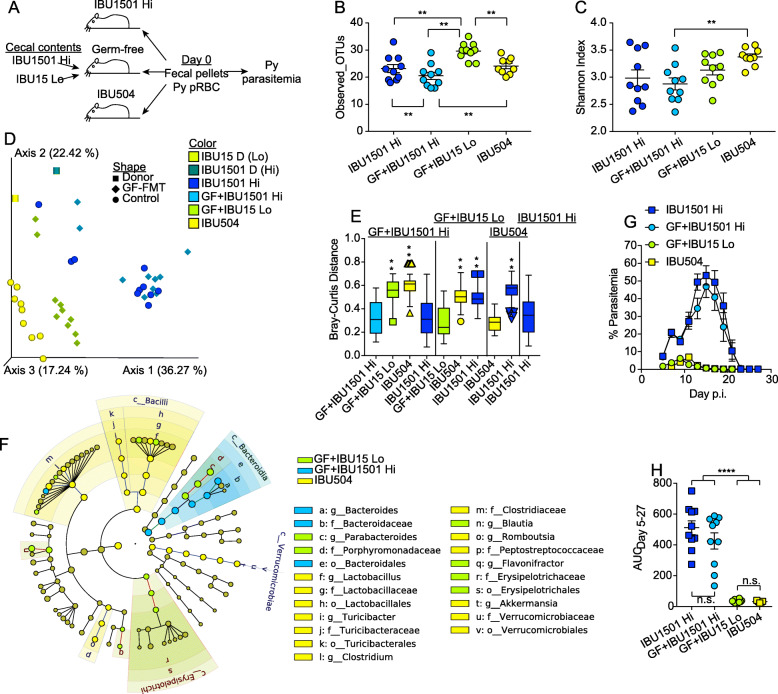
Shift in gut bacteria populations within Taconic C57BL/6N production suite causes differential severity of malaria. **a** Schematic of the experimental design. IBU1501 Hi = shipment of IBU1501 mice post-2017, IBU15 Lo = IBU15 mice from same shipment on August 2016 as reported in Fig. 1a, b. The cecum from the August 2016 mice (IBU15 Lo) had been removed and stored at − 80 °C. Ceca microbiota from IBU1501 Hi and IBU15 Lo were gavaged to GF mice in SPF facility and after 1 week were infected with *P. yoelii* 17XNL. The experiments were independently performed twice. **b**, **c** Fecal bacterial populations were profiled with alpha diversity using Observed_OTUs (**b**) and Shannon index (**c**). Data (mean ± S.E.) were analyzed by the Kruskal-Wallis test. **d** The PCoA plot shows beta diversity using the Bray-Curtis distance. **e** Statistical significance of beta diversity. The box end depicts the lower and upper quartiles and the horizontal line inside the box is the median while points outside the whisker are outliers. The *Y*-axis shows the distance of IBUs on the *X*-axis to IBUs on the top of vertical columns. Statistical significance is compared between IBUs on top of vertical columns to IBUs on the *X*-axis by pairwise PERMANOVA with 999 permutations. **f** Cladogram shows differentially abundant bacterial taxa among different groups with respective node color identified using LEfSe analysis. The cutoff for the LEfSe method was *p* < 0.05 (Kruskal-Wallis test) with LDA score > 4. **g** Parasitemia of mice on the indicated day post-Py infection. **h** Parasitemia AUC days 5–27 from **f**. Data are cumulative results with 9–10 mice per group from two experiments with each symbol representing an individual mouse. Data (mean ± S.E.) were analyzed by one-way ANOVA with Tukey’s multiple comparisons test. **p < 0.01, ***p < 0.001, ****p < 0.0001

To determine whether a discrete temporal shift occurred in gut microbiota between December 2016 and February 2017 and caused the change in malaria severity within IBU15 mice, cecal contents from mice before and after this timepoint were transferred into germ-free mice. Cecal contents extracted from IBU15 mice in August 2016 (Fig. 1a) were collected and frozen at − 80 °C; of note, other mice from this shipment had low *P. yoelii* parasitemia (Fig. 1a). These are referred to as the “IBU15 Lo” cecal contents. In contrast, mice received from IBU1501 post-2017 are referred to as “IBU1501 Hi” mice. Germ-free mice were colonized with cecal contents from either IBU1501 Hi or IBU15 Lo mice. Fecal pellets were collected from germ-free mice colonized with IBU1501 Hi or IBU15 Lo as well as control IBU1501 Hi and IBU504 (low parasitemia control) mice on the day of *P. yoelii* infection (Fig. 3a).

Alpha and beta diversity analyses of fecal bacteria populations showed that GF+IBU15 Lo had different numbers of species and bacteria composition compared to GF+IBU1501 Hi (Fig. 3b–d). Of note, differences in the bacteria populations between the two ceca donors, which represent temporally different and phenotypically distinct (with respect to *P. yoelii* parasitemia) mice from the same IBU, were evident in the principal coordinates analysis (PCoA) plot (Fig. 3d, box symbols). Collectively, these results demonstrate that there was a change in gut bacteria within IBU15 mice after August 2016. As expected, fecal bacteria compositions in GF+IBU1501 Hi mice were similar to those in control IBU1501 mice but were significantly dissimilar to those in GF+IBU15 Lo mice and control IBU504 mice (*p* < 0.01; Fig. 3e left grouping). Likewise, there was a significant difference in bacteria compositions in GF+IBU15 Lo mice and control IBU1501 Hi mice (*p* < 0.01; Fig. 3e second from left grouping). Although bacteria populations from GF+IBU15 Lo and control IBU504 mice are relatively similar to one another compared to the GF+IBU1501 Hi and control IBU1501 mice, indicated by their close proximity to one another on the PCoA plot (Fig. 3d), the bacteria populations were also significantly different between GF+IBU15 Lo and control IBU504 (*p* < 0.01; Fig. 3e second from left grouping). Moreover, most of the differentially abundant bacterial taxa were present in IBU504, which is consistent with these mice originating from a completely different facility (Fig. 3f). In support of the hypothesis that changes in gut bacteria composition in IBU15 mice caused the change in *P. yoelii* parasitemia, germ-free mice colonized with cecal content from IBU1501 Hi mice (GF+IBU1501 Hi) displayed high parasitemia similar to the control IBU1501 Hi mice, while germ-free mice colonized with cecal content from IBU15 Lo mice (GF+IBU15 Lo) had low parasitemia similar to control IBU504 mice (Fig. 3g, h). These data provide compelling evidence that a shift in gut microbiota in mice within the IBU15 production suite at the juncture of 2016 and 2017 profoundly altered the severity of malaria in these mice. It is currently unclear whether the observed change in gut bacteria (Fig. 3d–f) was responsible for the change in malaria severity or whether changes in other gut microbiota constituents (viruses, fungi, archaea, etc.) also impacted the severity of malaria.

### Differences in gut microbiota in Taconic mice impact reproducibility of additional murine model systems

**Fig. 4:**
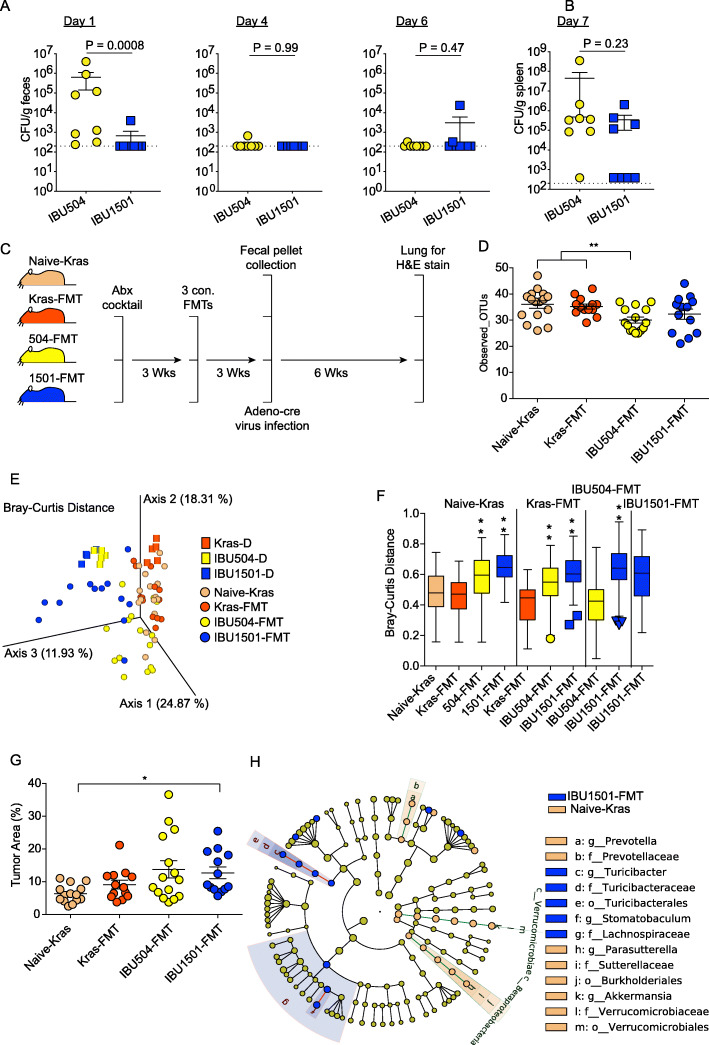
Difference in Taconic gut bacteria populations impacts reproducibility in infectious (*Salmonella*) and non-infectious (lung tumor) disease models. **a** C57BL/6N mice from the indicated IBUs at Taconic were infected with *S. enterica* serovar Typhimurium. *S. enterica* serovar Typhimurium bacterial burden was determined by measuring the number of colony-forming units (CFU) per gram of feces (**a**) or spleen (**b**) on the indicated day post-infection. Each symbol represents an individual mouse. Data (mean ± S.E.) are cumulative results with 7–8 mice per group from two experiments and were analyzed by the Mann-Whitney test. **c** Schematic of the lung tumor model. Two independent experiments were conducted with *n* = 8 per group. Kras mice were treated with antibiotics, received fecal microbiota transplantation (FMT) from naïve mice having low or high Py parasite burden, and infected with Adeno-cre virus for the spontaneous development of lung tumor. **d**–**f** Fecal pellets were collected and subjected to 16S rRNA gene sequencing. Data were analyzed at 19,000 sequencing depth per sample. **d** Alpha diversity using Observed_OTUs. Data (mean ± S.E.) were analyzed by the Kruskal-Wallis test. **e** The PCoA plot shows beta diversity using the Bray-Curtis distance. **f** Bray-Curtis dissimilarity index between indicated samples. The box end depicts the lower and upper quartiles and the horizontal line inside the box is the median while points outside the whisker are outliers. The *Y*-axis shows the distance of IBUs on the X-axis to IBUs on the top of vertical columns. Statistical significance is compared between IBUs on top of vertical columns to IBUs on the X-axis by pairwise PERMANOVA with 999 permutations. **g** Lung tumor burden in different groups of Kras mice as shown in **c**. Data (mean ± S.E.) were analyzed by the Kruskal-Wallis test and post hoc analysis with Dunn’s test. **h** Cladogram shows differentially abundant bacterial taxa between two IBUs having significantly different lung tumor burden with respective node color calculated using LEfSe analysis. The cutoff for the LEfSe method was *p* < 0.05 (Kruskal-Wallis test) with LDA score > 4. *p < 0.05, **p < 0.01, ***p < 0.001

These results led to the hypothesis that other model systems may also have been impacted by the change in gut microbiota within IBU15 mice. To test this possibility, mice were obtained from Taconic rooms IBU504 and IBU1501. Of note, this is not a direct comparison of Taconic IBU15 mice before and after the juncture of 2016 and 2017 that profoundly altered the severity of malaria in these mice owing to limitations in stored ceca samples from IBU15 mice pre-2017 (temporal shifts). The effects of different gut microbiota composition between IBUs (spatial change) were tested on two disease models. Following *Salmonella enterica* serovar Typhimurium infection, mice from Tac IBU504 had higher levels of *S. typhimurium* in feces on day 1 post-infection compared to Tac IBU1501 mice, while no differences were detected on days 4 and 6 in the feces or in the spleen on day 7 post-infection in these three groups of mice (Fig. 4a, b).

A similar trend was observed in LSL-Kras^G12D^ (Kras)-driven lung adenocarcinoma. Kras mice were treated with an antibiotic cocktail to deplete native microbiota. Mice were then gavaged with cecal microbiota from IBU504 (low parasitemia) and IBU1501 (high parasitemia) to recapitulate differential gut microbiotas between Taconic facilities. Finally, the mice were infected with Adeno-Cre virus to induce spontaneous lung adenocarcinoma (Fig. 4c and Additional file 2, Fig. S2A). Alpha and beta diversity (Bray-Curtis and weighted UniFrac) analyses showed no difference in gut bacterial composition between naïve Kras (Naïve-Kras) mice and Kras mice that received naïve Kras fecal microbiota (Kras-FMT) (Fig. 4d–f and Additional file 2, Fig. S2B, G, and H). Yet, beta diversity measured using Jaccard distance and unweighted UniFrac distance showed minor but significantly different gut bacterial composition between Naïve-Kras and Kras-FMT (Additional file 2, Fig. S2C-F). Kras mice that had IBU504 and IBU1501 fecal microbiota transplant (IBU504-FMT and IBU1501-FMT, respectively) had distinct gut microbiota composition compared to other groups (*p* < 0.01) (Fig. 4e, f and Additional file 2, Fig. S2C-H). An analysis of lung tumor burden demonstrated that only 1501-FMT developed significantly higher lung tumor burden compared to the other groups (*p* < 0.05, Fig. 4g). Further analysis demonstrated that naïve-kras mice (i.e., lower lung tumor burden) were associated with *Akkermansia*, *Prevotella*, and *Parasutterella* while 1501-FMT (i.e., higher lung tumor burden) were associated with *Turibacter* and *Stomatobaculu*m bacterial genera (Fig. 4h). Thus, differences in Taconic mouse gut microbiota sourced from different colonies also impacted the development of lung tumors. DSS-induced colitis has been shown to be impacted by gut microbiota [29], and minor differences were also noted between IBU504 and IBU1501 mice treated with DSS in body weight loss, though these differences were less pronounced (Additional file 3, Fig. S3). Although these latter mouse models do not exhibit as drastic differences like those seen between Taconic mice infected with *P. yoelii*, the collective data demonstrate that changes in gut microbiota, even within a specific vendor, can impact experimental reproducibility and represent a critical variable of which the scientific community needs to be aware.

## Discussion

To our knowledge, this is the first study to systematically report a shift in gut microbiota within genetically identical mice ordered from the same production suite from a specific vendor over time and the effect this shift in gut microbiota had on an experimental model system. Although this unexpected shift in gut microbiota negatively impacted our murine malaria model system, it is important to stress that it is abundantly clear that commercial providers of mice implement robust standard operating procedures to provide the scientific community with mice that are as homogenous over time as is technically and practically feasible. This is evident by the reproducibility of many model systems from many labs over time. Indeed, we have observed consistent phenotypes in *P. yoelii* parasite burden from multiple vendors for the 7+ years that we have studied the impact of gut microbiota on the severity of malaria. Still, the results presented in this report highlight that despite these rigorous practices, gut microbiota in mice can change within commercial vendors and that these changes can have profound implications.

It is clear based on the exponential growth in gut microbiota research over the past decade and the ever-increasing number of diseases and host processes that are impacted by gut microbiota that properly controlling for variations in gut microbiota can no longer be avoided. How should the scientific community respond to these realities? This likely involves responses by both commercial vendors and scientists. Regarding commercial vendors, they should raise the awareness and responsibility to at a minimum continue to provide transparency on the realities and limitations of commercial animal production. Commercial animal suppliers produce animals utilizing processes that prioritize the maintenance of high genetic quality and health standards [30, 31]. In order to provide animals that are at minimum specific pathogen free, vendors have exclusion criteria that stock are maintained against. Through processes developed to exclude organisms, it is likely that microbial diversity is restricted to varying degrees across all providers based on the specific health standard and husbandry practices used. To prevent genetic drift, periodic genetic refreshment is required whereby breeder animals are replaced using foundation colony stock with pedigreed genetics. This process also provides an opportunity to alter the microbial community of a mouse colony maintained under barrier conditions. It also suggests that a system of uniform health standards/profiles should be implemented across vendors to better support the scientific community. Animals of higher health status are generally housed in more controlled environments such as individually ventilated caging (IVCs) or even isolators. Furthermore, some commercial providers are developing services to generate and maintain gnotobiotic mouse models that can be utilized to address concerns relating to reproducibility and predictability. These options aid in reducing some microbiota shifts, but do not eliminate the possibility of change. Another possibility, albeit a more drastic measure, is for commercial vendors to routinely perform gut microbiota analysis of mice within production rooms and communicate the results of those analyses to the scientific community in a clear and meaningful way. This would introduce many challenges including the feasibility of such an undertaking, the frequency of gut microbiota analysis, and how to communicate those results in a manner that is meaningful to a non-microbiome expert.

Regarding a response from scientists, at the very least, these results emphasize the importance of reporting not only where mice were purchased from, but also the barrier room where those mice originated from. It is possible that many researchers are unaware of the numerous locations within a single vendor from which their mice may be originating, and if they are consistently coming from the same barrier room over time. Researchers also need to be mindful of the housing and husbandry methods in their own facilities. Publishers could help facilitate the consistent and uniform communication of mouse husbandry details through the inclusion of a mouse husbandry reporting standard that includes vendor name, mouse strain, barrier room, and institutional husbandry (type of water, diet details, bedding, light cycle, etc.). Implementation of this reporting in manuscripts will provide critical information to the scientific community that may improve experimental reproducibility within a lab over time, as well as the efforts of others to repeat the published studies. Do the realities highlighted in this report mean that researchers need to freeze feces from study animals and associate the next study cohort with the same microbiota? While it may sound drastic, it may be what is needed to generate reproducible data in cases where gut microbiota are particularly impactful to study outcomes.

## Conclusion

These data demonstrate that gut microbiota can undergo profound changes in commercial laboratory mice within specific vendor barrier rooms that have the potential to alter experimental reproducibility. Shared responsibility between vendors and scientists will allow more reproducible microbiome experiments. Vendors should provide scientist with mice gut microbiome profile in a timely manner. Scientist should provide all the necessary details regarding the source of mice including isolated breeding unit. In sum, we hope that introduction and application of these practices, to some extent, will re-build confidence of both the public and scientists themselves, in the scientific process and our endeavors to use biomedical research to make scientific advancements.

## Methods

### Mice

Female 6-week-old C57BL6/N mice were purchased from the indicated barrier rooms at Taconic Biosciences (Tac) (Hudson, NY) and allowed to acclimate for 1 week before collecting fecal pellets or cecal content and starting infections. Mice were kept on NIH-31 Modified Open Formula Mouse/Rat Irradiated Diet (Envigo item#; 7913, Huntingdon, UK) and autoclaved, non-acidified, reverse osmosis water. Mice were kept on a 12-h light/dark cycle from 6 AM to 6 PM and 6 PM to 6 AM, respectively. All animal handling and experimentation were reviewed and approved by the University of Louisville Institutional Animal Care and Use Committee based on the recommendations of the Guide for the Care and Use of Laboratory Animals of the National Institutes of Health. Mice were euthanized by carbon dioxide asphyxiation.

### *Plasmodium* infection

C57BL/6N mice were injected intravenously with 1 × 10^5^ red blood cells (RBCs) infected with *Plasmodium yoelii* 17XNL diluted in 200 μL of saline, passaged from donor infected mice. Parasitemia, the percentage of infected red blood cells, was monitored using flow cytometry beginning on day 5 p.i. and continuing every other day until clearance of the parasite. Blood was collected from the tails of infected mice. From each mouse, ~ 5 μL of whole blood was diluted in 100 μL of cold PBS, fixed in 0.00625% glutaraldehyde, and then stained. The staining panel included CD45.2-APC (clone 104; Biolegend, San Diego, CA), Ter119-APC/Cy7 (clone TER-119; Biolegend, San Diego, CA), dihydroethidium (MilliporeSigma, St. Louis, MO), and Hoechst 33342 (MilliporeSigma, St. Louis, MO); samples were then resuspended in flow cytometry buffer and analyzed. Infected red blood cells were identified by first gating on single cells followed by gating on Ter119^+^CD45.2^−^ red blood cells. Infected red blood cells were identified as dihydroethidium^+^Hoechst 33342^+^.

### Colonization of germ-free mice

Genetically identical germ-free (GF) C57BL/6 mice were ordered from Taconic Biosciences (Hudson, NY). GF mice were gavaged with cecal content from the indicated donor inside a laminar flow hood. Cecal content, either previously frozen or freshly extracted, was extruded out of the ceca and gently mixed for ~ 30 s in 5 mL 0.9% sterile saline (Teknova, Hollister, CA). Each mouse received 200 μL of diluted cecum material. After gavage, mice were conventionally housed as described above.

### *Salmonella* infection

*Salmonella enterica* serovar Typhimurium 14028 was grown overnight in Luria-Bertani (LB) medium (BD, Sparks, MD) with carbenicillin (50 μg/mL; Corning, Corning, NY) on a shaking rack at 200 RPM at 37 °C. One hundred microliters of overnight growth was transferred to 10 mL LB medium and grown to OD_600_ = 0.1. The subculture was washed once with 1X phosphate-buffered saline (PBS), and colony-forming unit (CFU) was adjusted to ~ 5 × 10^5^ CFU/200 μL in 1X PBS. CFU of inoculum was counted before and end of gavage to see the effect of static container sitting in the hood. No statistical difference in CFU of inoculum was seen. Each mouse was gavaged with 200 μL of inoculum around 2 PM for both trials. Inoculum gavage in IBU504 mice was followed by 1BU1501 mice for both trials. For *Salmonella* load in feces, one or two fresh fecal pellets were collected on indicated days in a sterile cryogenic vial on ice, weighed, homogenously mixed in 1X PBS, and plated on Brilliant Green agar (Sigma-Aldrich, St. Louis, MO) with carbenicillin (50 μg/mL). The plates were incubated for 18 h, and pink colonies were counted. For *Salmonella* load in the spleen, mice were euthanized at the end point (day 7 post-infection) and the spleen was harvested aseptically in 3 mL sterile 1X PBS and weighed. Spleens were homogenized and plated on LB agar plates with carbenicillin (50 μg/mL). Plates were incubated overnight, and colonies were counted.

### Kras-driven lung adenocarcinoma

LSLKras (B6.129S4-Krastm4Tyj/J) mice were purchased from The Jackson Laboratory (Bar Harbor, ME) and bred in a specific pathogen-free facility at the University of Louisville School of Medicine. Eight mice were allocated to each group (total 4 groups). Three groups of mice received an antibiotic cocktail (ampicillin (Sigma-Aldrich, St. Louis, MO), gentamicin (Corning, Corning, NY), metronidazole (Spectrum, Gardena, CA), and neomycin (Goldbio, St. Louis, MO) at concentration of 0.5 mg/mL, and vancomycin (VWR Chemicals, Sanborn, NY) at 0.25 mg/mL in drinking water for 3 weeks. Drinking water was changed weekly. Antibiotic treatment was ceased followed by fecal microbiota transplants (FMT) on 3 consecutive days. Fecal pellets were collected from each mouse prior to adenoviral-cre (Viral Vector Core Facility, University of Iowa) infection, and lung histological slides were done as described by Li et al. [32]. Briefly, adenoviral infection was performed intranasally in anesthetized mouse with isoflurane at 2.5 × 10^7^ PFUs per mouse in two 30-μL installations. Mice were housed as described above for 6 weeks to allow for the development of spontaneous lung adenocarcinoma. After 6 weeks, lungs were harvested for histological slides to quantify the tumor load. Briefly, lungs were harvested and infused with 10% neutral buffered formalin (Leica Biosystems) for 18 h, embedded in paraffin, and serially sectioned. Six representative sections were taken throughout the lung to provide representative samples and stained with H&E for microscopic analysis.

### Histological analysis

Image sections were captured with the Aperio ScanScope XT Slide Scanner (Aperio Technologies, Vista, CA) system with a × 20 objective, and tumor burden was quantified with QuPath v0.1.2 software as described in the QuPath manual for H&E stain [33]. Briefly, the image file was opened as image type H&E stain. Random trees classifier was interactively trained to distinguish between healthy and hyperplasia at the cellular level. The same classifier was used for all the slides. The results from QuPath were comparable to the manual score using Spectrum WebScope (Spectrum version 10.1.5.2028). Percent tumor area (tumor burden) was calculated as the ratio of tumor area to total lung area per section, and all sections from a single mouse were averaged to get tumor area per mouse.

### DSS-induced colitis

Mice from Tac IBU504 and Tac IBU1501 were administered 3% colitis grade DSS (MP Biomedicals, Solon, OH) in drinking water for 6 days to induce acute colitis, followed by cessation of treatment and return to conventional water for a 14-day recovery period. Control mice were on conventional water for the duration of the experiment. All mice were monitored for mortality and weight throughout treatment and recovery periods. The ceca and colon were harvested at the end of the experiment from control and DSS-treated mice. Fat was removed from colons followed by length measurements. Colons were then washed with 1X PBS followed by weight measurements.

### Gut microbiota analysis

Mouse fecal pellets and cecal contents were collected and flash frozen in liquid nitrogen followed by storage at − 80 °C. DNA was extracted using the QIAamp PowerFecal DNA kit (QIAGEN, Germantown, MD) according to the manufacturer’s instructions. DNA samples were shipped overnight on ice to the Genome Technology Access Center at Washington University (GTAC, St. Louis, MO) for sequencing and analysis using the Multiple 16S Variable Region Species-Level IdentificatiON (MVRSION) algorithm [34]. Observed taxonomic unit (OTU) table and phylogenetic tree file generated by MVERSION were imported inside QIIME2 [35] for visualization and statistical analyses. Alpha and beta diversity analyses for different IBUs (Fig. 2) and germ-free experiment (Fig. 3) were performed at 23000 sequencing depth per sample, and for lung adenocarcinoma (Fig. 4 and Additional file 2, Fig. S2), these were performed at 19000 sequencing depth per sample at the species level.

### Statistical analysis

Statistical analyses were performed using GraphPad Prism 7 software (GraphPad Software, La Jolla, CA, USA). For all analyses, the alpha was set at 0.05. For area under the parasitemia curve (AUC) analyses, the trapezoidal rule was used for Eq. (1):

1 $$ {\mathrm{AUC}}_{\left(t1\hbox{-} t\hbox{-} \mathrm{last}\right)}=\Sigma\ \left({p}_i+{p}_{i+1}\right)\times \left({t}_{i+1}-{t}_i\right)/2 $$

where “*p*” is the percent parasitemia at the designated time point “*t*” [36]. Specific statistical tests are described in the figure legends.

Differentially abundant bacterial taxa were identified using the linear discriminant analysis (LDA) effect size (LEfSe) method [37]. The cutoff values were *p* < 0.05 (Kruskal-Wallis test) with LDA score > 4.

## Supplementary information

**Additional file 1: Figure S1.** Bacterial beta diversity analysis between mice from different Taconic IBUs. Same samples and analysis as in Fig. 2. PCoA plot shows beta diversity using Jaccard distance (**A**), Unweighted (**C**) UniFrac and (**E**) Weighted UniFrac distance and their statistical significance is shown by (**B**), (**D**) and (**F**), respectively. Box end depicts lower and upper quartile and horizontal line inside box is median while points outside whisker are outliers. Y-axis shows distance of IBUs on X-axis to IBUs on the top of vertical columns. Statistical significance is compared between IBUs on top of vertical columns to IBUs on the X-axis by pairwise PERMANOVA with 999 permutations. * = *p* < 0.05, ** = *p* < 0.01.

**Additional file 2: Figure S2.** Effect of differential gut microbiota from Taconic IBUs on lung tumor model. **A**, Parasitemia of mice from IBU504 and IBU1501 shipments that were used as donor for fecal microbiota transplantation in naïve Kras mice. **B-H**, Gut microbiota composition of Kras mice that received distinct gut microbiota as described in Fig. 4. **B**, Alpha diversity using Shannon index. Data (mean ± S.E.) were analyzed by Kruskal-Wallis test. PCoA plot shows beta diversity using Jaccard distance (**C**), Unweighted UniFrac (**E**) weighted UniFrac distance (**G**); and statistical significances are shown by (**D**), (**F**), (**H**), respectively. Box end depicts lower and upper quartile and horizontal line inside box is median while points outside whisker are outliers. Y-axis shows distance of IBUs on X-axis to IBUs on the top of vertical columns. Statistical significance is compared between IBUs on top of vertical columns to IBUs on the X-axis by pairwise PERMANOVA with 999 permutations. * = *p* < 0.05, ** = *p* < 0.01. **I**, Representative H&E section of lung from naïve-Kras and IBU1501-FMT groups analyzed using QuPath. Green is healthy area while red is hyperplastic area.

**Additional file 3: Figure S3**. Differences in Taconic gut microbiota had minimal impact on DSS-induced colitis. **A**, Schematic of experimental design. Mice from IBU504 and IBU1501 received 3% DSS in drinking water for 6 days and switched to regular water for recovery. Mice were monitored for weight and mortality till the end of recovery period (14 days) and colon collected at the end. *N* = 5 for control and *N* = 10 for DSS treated mice. **B**, Percent survival. Statistical analyses were performed with Log-rank (Mantle-Cox) test. **C**, Weight gain. Unpaired t-tests were used compare % of initial body weight at each time point between the DSS treated and control mice. Colon weight (**D**) and colon length (**E**) of surviving mice at the end of recovery period. Statistical analyses were performed with ordinary one-way ANOVA followed by Tukey’s multiple comparisons test. * = *p* < 0.05, ** = *p* < 0.01, *** = *p* < 0.001, **** = *p* < 0.0001.

## Data Availability

Raw reads of 16S rRNA sequencing data is submitted to Sequence Read Archive (SRA) under the BioProject ID PRJNA636965 (http://www.ncbi.nlm.nih.gov/bioproject/636965).
