# Supplementary figures and images for: Temporospatial shifts within commercial laboratory mouse gut microbiota impact experimental reproducibility
Source: BMC Biol. 2020 Jul 3;18:83. doi: 10.1186/s12915-020-00810-7 (PMC7334859; doi:10.1186/s12915-020-00810-7)

Additional File Figure S1

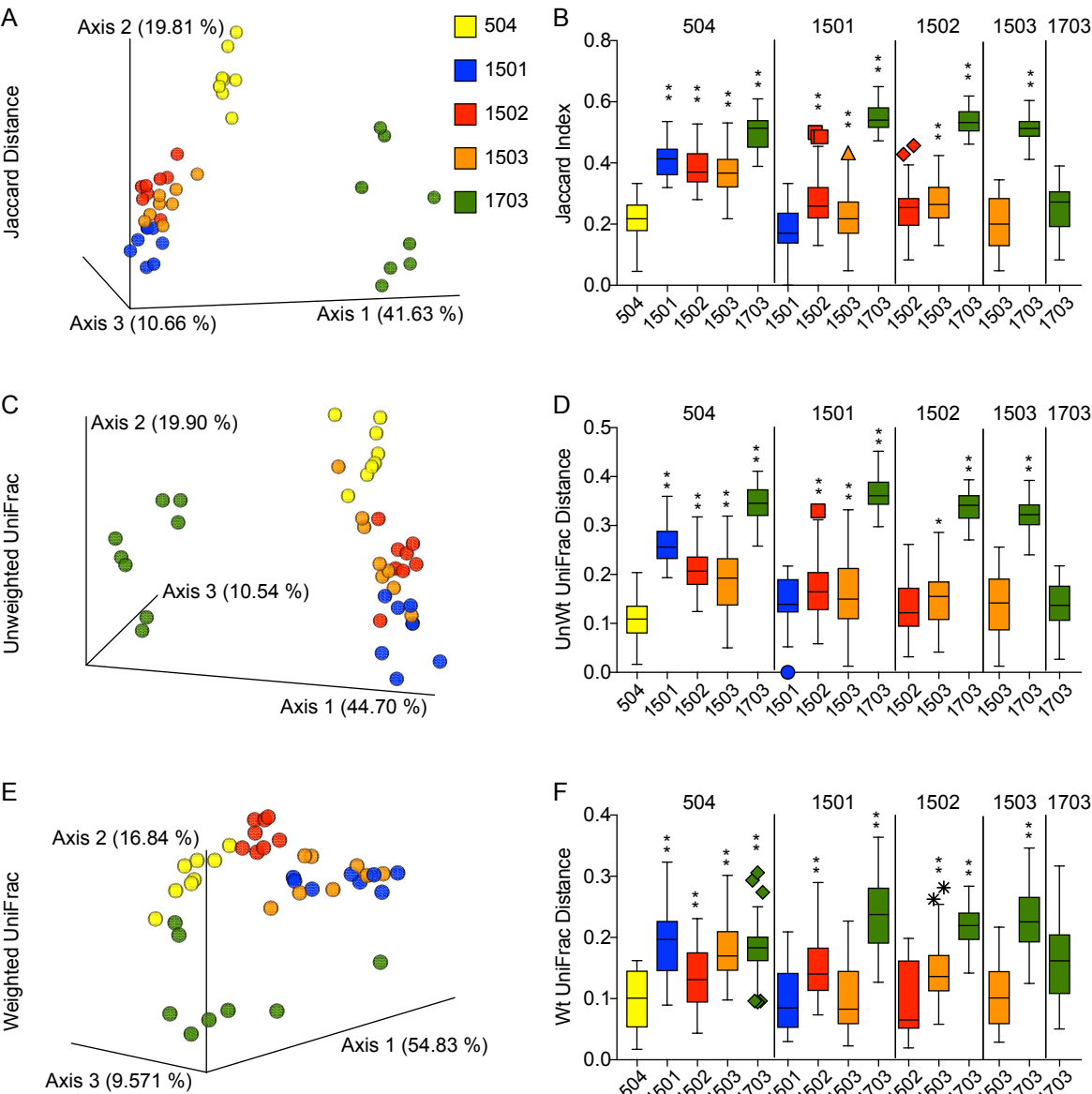

Supplement: Supplementary file 1 — Additional file 1: Figure S1. Bacterial beta diversity analysis between mice from different Taconic IBUs. Same samples and analysis as in Fig. 2. PCoA plot shows beta diversity using Jaccard distance (A), Unweighted (C) UniFrac and (E) Weighted UniFrac distance and their statistical significance is shown by (B), (D) and (F), respectively. Box end depicts lower and upper quartile and horizontal line inside box is median while points outside whisker are outliers. Y-axis shows distance of IBUs on X-axis to IBUs on the top of vertical columns. Statistical significance is compared between IBUs on top of vertical columns to IBUs on the X-axis by pairwise PERMANOVA with 999 permutations. * = p < 0.05, ** = p < 0.01. [file 12915_2020_810_MOESM1_ESM.pdf]

Additional File Figure S2

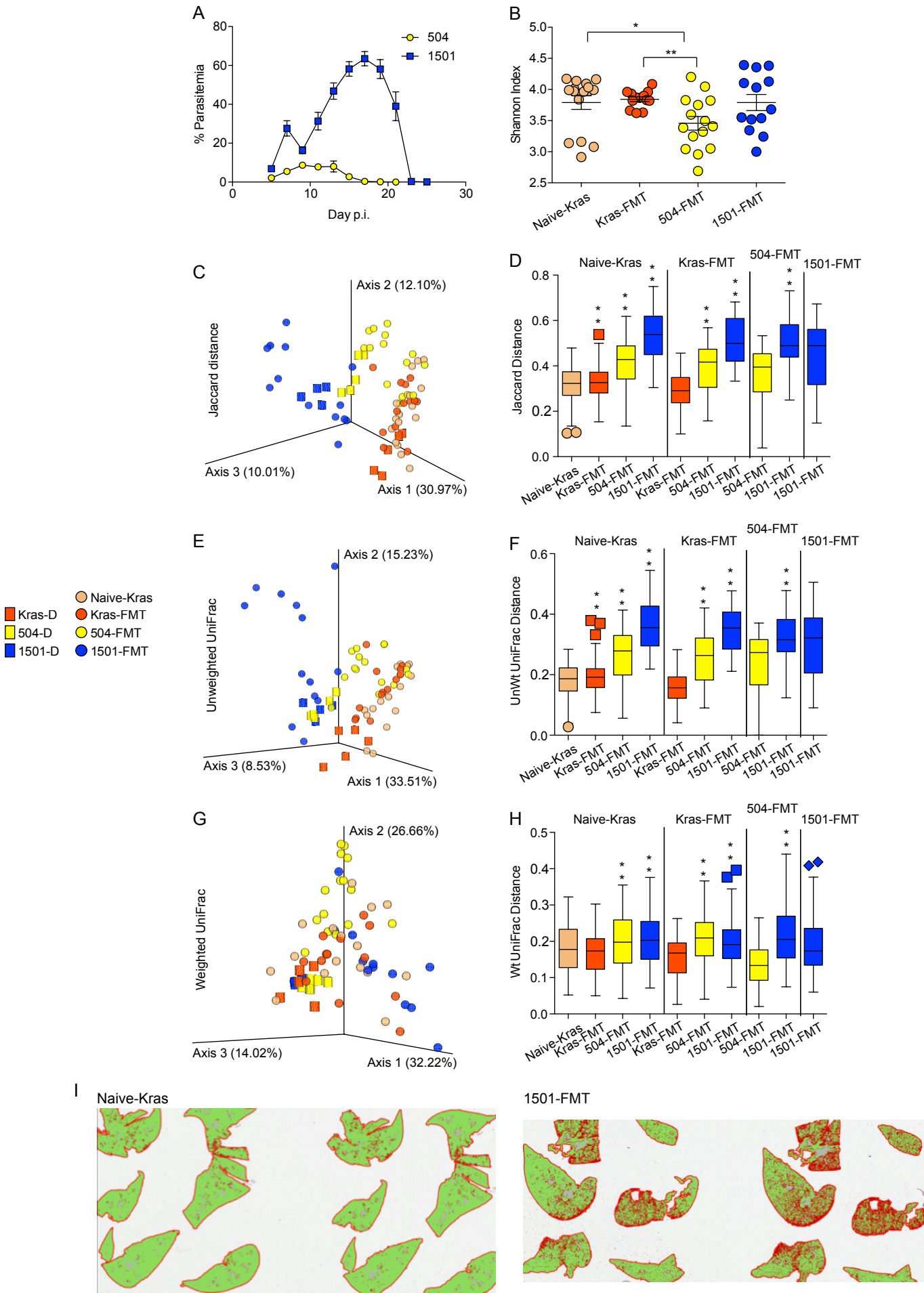

Supplement: Supplementary file 2 — Additional file 2: Figure S2. Effect of differential gut microbiota from Taconic IBUs on lung tumor model. A, Parasitemia of mice from IBU504 and IBU1501 shipments that were used as donor for fecal microbiota transplantation in naïve Kras mice. B-H, Gut microbiota composition of Kras mice that received distinct gut microbiota as described in Fig. 4. B, Alpha diversity using Shannon index. Data (mean ± S.E.) were analyzed by Kruskal-Wallis test. PCoA plot shows beta diversity using Jaccard distance (C), Unweighted UniFrac (E) weighted UniFrac distance (G); and statistical significances are shown by (D), (F), (H), respectively. Box end depicts lower and upper quartile and horizontal line inside box is median while points outside whisker are outliers. Y-axis shows distance of IBUs on X-axis to IBUs on the top of vertical columns. Statistical significance is compared between IBUs on top of vertical columns to IBUs on the X-axis by pairwise PERMANOVA with 999 permutations. * = p < 0.05, ** = p < 0.01. I, Representative H&E section of lung from naïve-Kras and IBU1501-FMT groups analyzed using QuPath. Green is healthy area while red is hyperplastic area. [file 12915_2020_810_MOESM2_ESM.pdf]

Additional File Figure S3

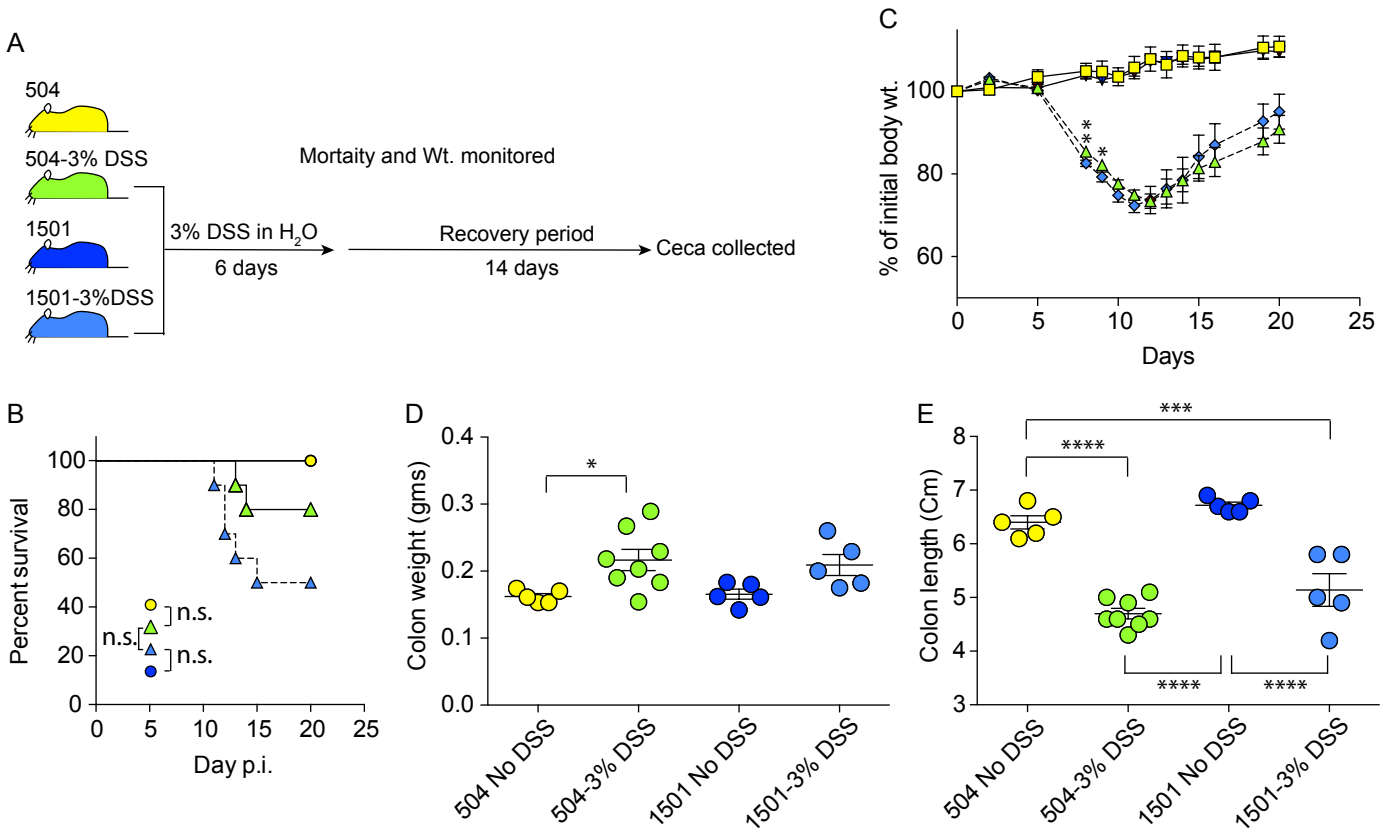

Supplement: Supplementary file 3 — Additional file 3: Figure S3. Differences in Taconic gut microbiota had minimal impact on DSS-induced colitis. A, Schematic of experimental design. Mice from IBU504 and IBU1501 received 3% DSS in drinking water for 6 days and switched to regular water for recovery. Mice were monitored for weight and mortality till the end of recovery period (14 days) and colon collected at the end. N = 5 for control and N = 10 for DSS treated mice. B, Percent survival. Statistical analyses were performed with Log-rank (Mantle-Cox) test. C, Weight gain. Unpaired t-tests were used compare % of initial body weight at each time point between the DSS treated and control mice. Colon weight (D) and colon length (E) of surviving mice at the end of recovery period. Statistical analyses were performed with ordinary one-way ANOVA followed by Tukey’s multiple comparisons test. * = p < 0.05, ** = p < 0.01, *** = p < 0.001, **** = p < 0.0001. [file 12915_2020_810_MOESM3_ESM.pdf]
